# How nurses’ moral competence can be supported: Findings from international focus groups with professionals

**DOI:** 10.1111/inr.13080

**Published:** 2024-11-27

**Authors:** Johanna Wiisak, Riitta Suhonen, Alessandro Galazzi, Chris Gastmans, Brian Keogh, Evridiki Papastavrou, Nikos Stefanopoulos, Alvisa Palese, Minna Stolt, the PROMOCON Consortium

**Affiliations:** ^1^ Department of Nursing Science University of Turku Turku Finland; ^2^ Turku University Hospital Turku Finland; ^3^ Wellbeing Services County of Southwest Finland Turku Finland; ^4^ Department of Medicine University of Udine Udine Italy; ^5^ Department of Public Health and Primary Care Centre for Biomedical Ethics and Law, KU Leuven Leuven Belgium; ^6^ School of Nursing and Midwifery, Trinity College Dublin Dublin Ireland; ^7^ Department of Nursing School of Health Sciences Cyprus University of Technology Limassol Cyprus; ^8^ Cyprus Nurses and Midwives Association Nicosia Cyprus; ^9^ Department of Nursing University of Patras Patra Greece; ^10^ Wellbeing Services County of Satakunta Pori Finland

**Keywords:** Focus groups, moral competence, moral development, nurses, nursing educators, nursing students

## Abstract

**Aim:**

To describe how nurses’ moral competence can be supported from the perspective of nurses, nurse managers, researchers, educators, and nursing students.

**Background:**

Moral competence is the capacity or ability of nurses to recognise one's own emotions of what is right or wrong, to reflect on these emotions, to make decisions, and to act in ways that bring the highest level of benefit to patients. Moral competence is part of professional competence. However, little is known about how nurses’ moral competence can be supported.

**Methods:**

A qualitative descriptive study design was applied. Stratified purposive sampling was employed and focus group discussions were conducted in Belgium, Cyprus, Finland, Greece, Ireland, and Italy in 2023. A total of 38 informants (5–8 per focus group) who were registered nurses or nursing students participated. The data were analysed using both deductive and inductive content analysis. The COnsolidated criteria for REporting Qualitative research were adhered to.

**Results:**

Seven themes were developed following analysis, which suggested that support for nurses’ moral competence can be located at individual‐relational, organisational, and societal levels. Several approaches and/or tools were also identified to support moral competence.

**Conclusion:**

Nurses’ moral competence could benefit from continuous support from colleagues, those in leadership positions, organisations and society. Practical tools and approaches can also successfully support nurses’ moral competence.

**Implications for nursing and health policy:**

Support for nurses’ moral competence forms a continuum from the beginning of nursing studies throughout nursing careers. Thus, educational interventions and training programmes are needed both at basic and continuous ethics education. There is also a need for investments in and development of strategies and regulations on ethics management in health systems, national‐ and international‐level ethics indicators for health policy‐making, and implementation of existing practices, interventions, and procedures in nursing practice.

## INTRODUCTION

Moral competence is fundamental to the provision of ethically high‐quality healthcare (European Commission, 2008; Lechasseur et al., 2018), which is essential to nurses in their daily practice (Kulju et al., 2016). Constant societal changes along with scarce resources, technological advances, increasing economic constraints, and conflicting values because of heavy prioritisation (National Academies of Sciences, Engineering, and Medicine [NASEM], 2021; United Nations, 2023) require nurses to develop their moral competence continuously. However, unmet needs and a lack of opportunities for nurses to develop their moral competence have been reported (Poikkeus et al., 2020), leading to increased moral distress and turnover, worsening the workforce shortage in healthcare (NASEM 2021). Thus, support for moral competence is required to enable the moral growth of nurses and the provision of ethically high‐quality and sustainable care for patients. Despite this, there is a dearth of empirical research which focuses on how to support nurses’ moral competence (see Koskenvuori et al., 2019; Wiisak et al., 2024). As nurses’ moral competence can be supported in both education and clinical environments, this study takes a broad perspective on this support as perceived by nurses and nursing students.

## BACKGROUND

Moral competence is the capacity or ability of nurses to recognise their own emotions (affective dimension) of what is right or wrong, to reflect on these emotions, to make decisions (cognitive dimension), and to act in ways that bring about the highest level of benefit for patients (behavioural dimension) (Jormsri et al., 2005). Formed in a social context, moral competence occurs as ethical performance based on ethical sensitivity, knowledge of ethics, and ethical decision‐making (Poikkeus et al., 2020). In this study, nurses’ moral competence is defined in terms of knowledge (knowing), perceptions (seeing), reflection, deliberation, and performance (being and acting as a professional nurse) (Kulju et al., 2016). Support has been described as one of the preconditions for the manifestation of moral competence (Kulju et al., 2016; Robichaux et al., 2022).

Nurses’ moral competence can be supported in various ways at all levels from an individual nurse (micro) through the organisational (meso) to the societal (macro) level (Wiisak et al., 2024). At the individual level, nurses need support to comply with ethical values and principles, professional codes of ethics, and legislation (Poikkeus et al., 2020). In addition, developing ethical reflection requires support, in both individuals’ self‐reflection and their reflection with others (Koskinen et al., 2022; Wiisak et al., 2024). As a form of mutual interaction, support and feedback from and role modelling by superiors and peers have been identified as important for developing moral competence (Koskenvuori et al., 2019; Wiisak et al., 2024). Organisational support for moral competence with supporting ethics infrastructures and ethics management strategies are needed but often missing despite leaders’ responsibility for implementing these, thereby reducing the risk of unethical wrongdoing (Poikkeus et al., 2020). Moreover, by creating a supportive environment, transparent reflection on ethical issues is possible, thus reducing the risk of negative responses and increasing engagement in ethical activity (Borhani et al., 2010).

Some approaches and tools to support nurses’ moral competence have been identified in the literature. At the individual level, in nursing education and continuous ethics education, moral competence can be supported by various learning and teaching approaches such as interactive teaching, e‐learning, problem‐based, and simulation‐based learning (Andersson et al., 2022; Lechasseur et al., 2018). Some tools with self‐assessment of one's own moral competence or guidance for joint reflection have been developed to support nurses dealing with moral challenges in clinical practice (Kröger et al., 2024; van Schaik et al., 2023). At the organisational level, nurses’ moral competence can be supported by establishing ethics committees and arranging regular ethics training and meetings to deal with ethical issues (Maluwa et al., 2021). Moreover, leaders are in a key position to support nurses’ moral competence throughout their nursing career, starting with recruitment reviews and continuing with regular performance appraisals (Maluwa et al., 2021; Poikkeus et al., 2020). At the societal level, nurses’ moral competence can be supported through legislation and values in society, on which morally competent nurses can rely on their ethical reasoning and decision‐making (Poikkeus et al., 2020; Wiisak et al., 2024). Given the complexity of health systems in different countries, which are in a continuous state of transition, conflicting values and ethical issues are frequently encountered; thus, professionals need constant support to prepare for the ethical issues and challenges they encounter (NASEM 2021).

## AIM OF STUDY

The study aimed to describe how nurses’ moral competence can be supported from the perspective of nurses, nurse managers, researchers, educators, and nursing students. The study sought to answer the following research questions: (I) How can nurses’ moral competence be supported? and (II) What approaches and tools can be used to support nurses’ moral competence?

## METHODS

### Research design

A qualitative descriptive study was applied using data collected with focus group discussions. This study is part of an international research project PROmoting a MOrally COmpetent Nurse (PROMOCON) involving six countries: Belgium, Cyprus, Finland, Greece, Ireland, and Italy. Data were, therefore, collected through focus group discussions in each of these countries. Focus groups were considered suitable as they capture active interactions between the informants who share their perspectives and lived experiences. Thus, they provide a rich and heterogeneous understanding from the perspective of both those whose moral competence is supported and the key persons who support it. (Liamputtong, 2016). The study has been reported according to the COnsolidated criteria for REporting Qualitative research (COREQ) checklist (Tong et al., 2007) (see Supporting Information File S1).

### Sample and setting

Stratified purposive sampling was employed to involve the informants in each country. This sampling method was adopted to select those informants that potentially provide the best information regarding the research topic (Liamputtong, 2016). Researchers involved informants via email or phone including those who were (1) professional nurses who have a nursing qualification (registered nurse); (2) acting as professional nurses, nurse managers, researchers or educators; or (3) undergraduate (e.g., registered nurse students) or postgraduate nursing students (e.g., master and doctoral students). Professionals were excluded if they (1) were not willing to participate or (2) not active in their professional or student role.

### Data collection

The data were collected with focus group discussions in each country between April and July 2023, using the native language of each country. A predefined interview protocol for data collection was followed (see Supporting Information File S2; Joyce, 2008). Based on previous research (Koskenvuori et al., 2019; Kulju et al., 2016), the protocol was prepared by researchers in Finland, complemented by others, and the final version was approved by the international research team. The interview protocol was pilot‐tested with one focus group in Italy. Three informants were registered nurses (two master's students and one nurse educator) who were invited directly to participate the piloting in one university. Piloting lasted around one and a half hours, and no changes were made to the protocol.

Informants were asked to describe how and with what approaches or tools nurses’ moral competence can be supported. The focus groups were conducted in private rooms in the departments of the universities where the researchers work. Each focus group included a moderator who was responsible for leading the discussion. In addition, one or two observers were present to make field notes from the discussions and to ensure that the interview protocol was complied with. The number of informants in the focus groups ranged from five to eight. Each focus group lasted approximately two hours. All focus group discussions were audio‐recorded, and memos were written by researchers during the discussions.

### Ethical considerations

The study was approved by the ethical committees of four countries: Cyprus (Ref No 2023.01.82), Greece (Ref No 29974), Ireland (Ref No 230501), and Italy (Ref No 33/2023). According to legislation in Belgium and Finland, ethical approval in this kind of study was not required; however, in Finland, the study was approved by the Internal Review Board (IRB 18042023) of the university. The study followed good scientific practice in each phase (All, European Academies [ALLEA], 2023). Each informant received written and oral information about the study purpose, data collection, confidentiality, and reporting of the results. Publication ethics (Committee on Publication Ethics [COPE], 2022) was adhered in reporting. At the beginning of each focus group, the purpose and data collection procedures were repeated, and informants could ask questions about the study. Each informant gave their written informed consent before the study. The General Data Protection Regulations (European Parliament and Council 2016/679) were adhered to, and a data management plan was in place.

### Data analysis and synthesis

The data were analysed using both deductive and inductive content analysis. Each audio‐recorded focus group was transcribed verbatim and analysed in the local language in each country. Two research questions guided the data analysis. Initially, the analysis was deductive, with the data sorted based on the research questions. The manifest content was coded and labelled, and the authentic quotations were linked to labelled textual expressions using the national language. Second, the coded and labelled expressions were translated into English using a data extraction sheet. Third, the country‐based coded data and included expressions were merged as one data set. Fourth, the data analysis was continued inductively by forming and labelling the common themes. The themes were worked on further within a specific research team including a team member from each country. Fifth, the group further condensed the preliminary themes into higher‐order themes that were well‐saturated, given the data collected from each country. The original quotations were kept when formulating the final themes to keep the analysis connected to the data for reporting. Authentic quotations that reflect the content are provided and marked with country‐level acronyms: Belgium (BE) Cyprus (CY), Finland (FI), Greece (GR), Ireland (IR), and Italy (IT).

### Rigour

To ensure rigour, the data collection process was conducted using a developed and agreed protocol. This ensured consistency across all countries when conducting the focus groups. This protocol was then used to support the data analysis once the data had been collected, to ensure that the coding and labelling of data were consistently applied using the same framework. Once the initial data analysis had been completed, they were translated into English, and a subgroup was formed to develop the emerging themes further. Care was taken to ensure that meaning was applied correctly, the translations were precise, and the concepts were explained clearly and without ambiguity. This was an iterative process that culminated in thick descriptions of the concepts. Frequent meetings were held with the team, and robust discussions were held to ensure that the themes developed were close to the data. This was further supported by using excerpts from the transcripts to highlight important points in the themes. The use of the protocol in each country acted as an audit trail, and the subgroup was able to establish a clear link from the raw transcribed data to the developed themes.

## RESULTS

### Description of the informants

A total of 38 informants participated, of whom 36 were registered nurses, and two were nursing students (Table 1). Eight informants were nursing educators. Of the nurses, most informants had a bachelor's or master's level education and two had a doctoral level education. Most of the informants were female, between 20 and 39 years old, with no or up to 19 years of work experience. The informants were from clinical practice, academic universities, or universities of applied sciences.

**TABLE 1:** Informants’ demographics stratified by focus group (total *n* = 38).

Focus group	BE	CY	FI	GR	IE	IT	Total	%
Number of participants	6	6	8	6	5	7	38	100
Gender								
Male	2	2	1	2	1	4	12	31.6
Female	4	4	7	4	4	3	26	68.4
Age (years)								
20–29	1	3	1	1	2	2	10	26.3
30–39	0	3	6	0	2	2	13	34.2
40–49	0	0	0	0	1	3	4	10.5
50–59	4	0	0	4	0	0	8	21.1
60–69	1	0	1	1	0	0	3	7.9
Highest nursing education								
Nursing student	0	0	2	0	0	0	2	5.3
Registered nurse (bachelor level)	4	4	3	4	2	4	21	55.3
Registered nurse (master level)	2	2	1	2	3	3	13	34.2
Registered nurse (doctoral level	0	0	2	0	0	0	2	5.3
Additional professional nursing qualification								
No	1	0	8	1	3	3	16	42.1
Yes	5	6	0	5	2	4	22	57.9
Community nursing	1	2	0	1	0	0	4	10.5
Bioethics	1	0	0	1	0	1	3	7.9
Mental health nursing	1	0	0	1	1	0	3	7.9
Geriatric nursing	1	0	0	1	0	0	2	5.3
Intensive care	0	2	0	0	0	0	2	5.3
Palliative care leadership	1	0	0	1	0	0	2	5.3
Physical activities science	0	0	0	0	0	1	1	2.6
Haemodialysis and organ transplantation	0	0	0	0	0	1	1	2.6
Health care management	0	0	0	0	0	1	1	2.6
Information Technology	0	1	0	0	0	0	1	2.6
Midwifery	0	0	0	0	1	0	1	2.6
Ophthalmology specialisation	0	1	0	0	0	0	1	2.6
Years of nursing experience								
0–9	1	4	7	1	4	3	20	52.6
10–19	2	2	1	2	1	2	10	26.3
20–29	0	0	0	0	0	2	2	5.3
30–39	3	0	0	3	0	0	6	15.8
Current position								
Clinical/bedside nurse	4	6	2	4	5	3	24	63.2
Head nurse	0	0	0	0	0	2	2	5.3
Nursing educator/teacher	2	0	2	2	0	2	8	21.1
Nursing researcher	0	0	2	0	0	0	2	5.3
Nursing student	0	0	2	0	0	0	2	5.3

Abbreviations: BE, Belgium; CY, Cyprus; FI, Finland; GR, Greece; IE, Ireland; IT, Italy.

### Supporting nurses’ moral competence

Seven themes were identified on support for nurses’ moral competence. Three of these themes were located at the individual‐relational level: (1) to give value to professional ethics, (2) to give value to others, and (3) to work on one‐self; two themes at the organisational level: (4) to ground the elements to deepen the ethical and moral field of the profession, (5) to sharpen the qualitative approach to care; and two themes at societal level: 6) to recognise the value of the nursing profession, and 7) to influence society (Table 2).

**TABLE 2:** Supporting nurses’ moral competence on individual‐relational, organisational, and societal levels.

Level	Themes	Subthemes
Individual‐relational level	To give value to professional ethics	Having an understanding of professional ethics
		Recognising the ethical element in every nursing activity
	To give value to others	Working effectively with others
		Considering of the opinions/points of views, perspectives of team members
		Creating synergy among colleagues
		Creating listening spaces
		Being critical
	To work on one‐self	Being an example
		Being proactive
Organisational level	To ground the elements to deepen the ethical and moral field of the profession	Creating a culture of ethics and morality
		Creating a strong multi‐professional team
	To sharpen the qualitative approach to care	Evaluation of services provided with indicators, including qualitative ones (e.g., the quality of care)
		Creating working conditions supporting ethical practice
Societal level	To recognise the value of the nursing profession	Being recognised by the community
		Integrating social values to nursing
	To influence society	Identifying a pathway for citizens to report concerns about ethical practice
		Increasing public awareness on the ethics of care and patients’ rights

The support for nurses’ moral competence at the individual‐relational level includes the individual nurse who works in a relational context with colleagues from different disciplines. At this level, the support of nurses’ moral competence consists of giving value to professional ethics, valuing others, and working on oneself.

Giving value to professional ethics consisted of understanding professional ethics and recognising the ethical element in every nursing activity. Valuing others included working effectively with others and considering different opinions/points of view, the perspectives of team members, the creation of synergy among colleagues, creation of listening spaces, and the ability to be critical. Working effectively with others entails mutual respect and responsibility in caring for patients. Collegial support for others in developing skills and behaviours in ethical practice, as well as daring to break down the routines and recognition of stressors that threaten ethical practice, were considered important.

*In my opinion, waiting for an organisation to take over a situation, I mean, we can in our own small way. That is in my opinion also the beauty of the profession from an ethical point of view. That is, the synergy between all: I help you in this, I share. (IT)*

Informants underlined the consideration of all team members’ opinions and points of view as recognising nurses’ opinions and reflecting the hidden curriculum shaping behaviour in practice.

*Experience in education is important, and so is knowledge of teaching methods/techniques. But there is also a kind of talent, some people have it and others don't, and it's about being able to transfer things to your students. (GR)*

Creating synergy among colleagues was considered important, particularly in transferring knowledge and providing educational opportunities, avoiding solution‐oriented practice and ensuring regular evaluation and feedback were central features of support.

*Sometimes nurses don't acknowledge ethical issues… they carry out tasks and don't question anything. They don't think about ‘I should do better’. (IR)*

Creating listening spaces for ethical reflective discussions by offering ethical support during the work‐day was considered important.

*Valuing time so that ethical reflection is part of work and working time. (FI)*

Being critical was seen as a central element in supporting moral competence, which includes whistleblowing, reporting unethical practices, questioning everyday actions, and asking the right questions.

*How do we decide and what do we prioritise now in our daily work? Through case studies, i.e. examples through experiential experiences, when you come to say, ‘This happened in our department today, what could we do to avoid it?’ The brainstorming of ideas in staff meetings, it is very important to understand our behaviours…. (CY)*

To support moral competence, nurses as individuals need to work on themselves and be an example, acting as role models to others, inspiring colleagues, and being proactive in implementing ethical decision‐making.

*That should not actually be limited to your own interaction with patients, that must be propagated and stimulated, and that must be a kind of movement… I mean, that must inspire other people to be able to provide caring care or tailored care, where you start from vulnerability and not …. (BE)*

To support the moral competence of nurses at the organisational level, there is a need to deepen the ethical and moral field of the profession and to sharpen the qualitative approach to care. Creating an ethically supportive culture and a solid multi‐professional team were underlined. These could be achieved through ethical support structures, an ethical climate, ethical experts, and non‐judgemental support in cases of ethical dilemmas.

*What can the healthcare organisation do? That's something that doesn't happen overnight, that's a long‐term process of paying attention to ethics. (BE)*

Moreover, the informants emphasised the qualitative approach to care to support nurses*’* moral competence. This meant having an evaluation of services provided with indicators, including qualitative ones (e.g., indicators to evaluate the quality of care) and creating working conditions that support ethical practice. The latter may contribute to making nurses’ roles visible, monitoring ethical stress, and promoting staff discussions as essential in supporting moral competence.

*The supervisors help nursing students with practical skills but also with developing a professional culture – help them to understand what it means to be a nurse. (GR)*

Support of nurses’ moral competence on a societal level involves the recognition of the value of the nursing profession and its influence on society. The recognition of the value of the nursing profession was seen as a basis of nursing care consisting of recognition by the community and integration of social values into nursing. The provision of societal‐level ethical guidelines and recommendations and policy‐making were underlined.

*This is a sign of acknowledgement of the citizens, of the people we have in charge, toward nurses. We heard so much with COVID, we were always talking about nurses. Now a little less. This is probably one of the most motivating factors for nurses, because they feel that their work, their profession is useful and recognized by people. So definitely the social recognition of the profession. (IT)*

Influencing society was seen as important in the promotion of nursing in society and, through that, also in support for moral competence; for example, identifying pathways for citizens to report ethical issues and increasing public awareness of the ethics of care and patients’ rights or increasing the attractiveness of the profession.

*Role of the media – how nursing and its critical issues are presented, how they are dealt with, highlighting good things alongside negative news – developing a non‐blaming climate, and society could be supportive rather than blaming. (FI)*

### Approaches and tools to support nurses’ moral competence

The informants identified many practical approaches and tools to support nurses’ moral competence in the educational, emotional, and practical fields (Table 3).

**TABLE 3:** Approaches and/or tools to support nurses’ moral competence.

Field	Approaches and/or tools
Educational	Education, also including interprofessional education
	Innovative teaching methods
	Internship education
Emotional	Tools for communicating and processing emotions (e.g., reflective diary)
	Creating spaces for sharing and reflecting
Practical	Providing a toolkit
	Spaces (physical, time, and promotion) for shared reflection
	Providing material resources

In the educational field, interprofessional education characterised by innovative teaching methods (e.g. simulation‐based teaching, case‐based teaching) as continuous or in internship education was suggested.

*Education about defining moral competence and mainly including examples for application to practice that is normative and applied to all care scenarios rather than seen as a separate entity. (IR)*

In the emotional field, tools for communication and processing emotions and for creating spaces for sharing experiences and reflecting were important approaches.

*Also good to experience some cases … after failure…, how to learn from them. (FI)*

In the practical field, providing an ethics toolkit, including different kinds of individual or organisational tools to promote moral competence, was suggested. The toolkit would include ethical protocols, checklists, guidelines, and job counselling.

*With the development of protocols and check‐lists within the clinical area, I believe that we can achieve two goals. Firstly, we will all work in the same way, whether you are a novice or experienced, and secondly, the check‐lists certify the protocols. (CY)*

## STUDY LIMITATIONS

Some of the limitations of this study concern the group dynamics. More precisely, the power relationships (e.g., between students and educators) and fluctuating nature of group discussions may have meant that some topics, insights, or individual perspectives may have been missed or remained hidden. However, as strengths, to overcome these, the focus group discussions followed a predefined standardised interview protocol and the moderators were instructed to lead the discussions. In addition, the moderators regularly inquired whether the informants had further ideas to elaborate on topics under discussion and ensured that everyone's voice was heard. This was also ensured by the observers following the focus group discussions.

## DISCUSSION

The study provided novel findings on how nurses’ moral competence can be supported as perceived by nursing students and nurses with different levels of education and working positions. Support for nurses’ moral competence occurs at the individual‐relational, organisational, and societal levels. Tools and approaches to support nurses’ moral competence are pragmatic and focus on the educational, emotional, and practical fields.

Ethical dilemmas and issues are confronted daily in nursing practice (Giannetta et al., 2021; Haahr et al., 2020). Therefore, preparedness for such situations requires continuous education (Robichaux et al., 2022) and integrated support systems (Wiisak et al., 2024). Although there is a debate in the literature on how to sensitise nursing students to the development of moral competence (Spekkink & Jacobs, 2021), little attention has so far been paid to how its development can be continuously supported. The levels of this expected support mirror those already identified at the individual nurse (micro) through organisational (meso) to societal (macro) levels (Wiisak et al., 2024) working integrated with each other.

At the individual‐relational level, support for moral competence is expected or enacted in daily nursing practice. At the individual‐relational level, support for moral competence is expected or enacted in daily nursing practice. Supporting moral competence requires not only an active role of the individual, but also interaction, human encounters, and socialisation processes. One of these processes is professional socialisation, which results in the growth of professionalism and professional identity through the internalisation of ethical knowledge and skills, attitudes, and values in everyday professional practice and behaviour, especially for nurses who have just entered the nursing profession (Shahr et al., 2019).

At the organisational level, several integrated systems are used to handle ethical issues (Park, 2012), including nursing boards, ethical committees, ethics rounds, and clubs where moral competence is promoted, controlled (through codes of ethics or conduct), or formed. However, they have not necessarily been implemented yet or are still fragmented and isolated interventions produced by different sources (Kaşıkçı & Yıldırım, 2024). This study highlighted the importance of deepening and sharpening the ethical and moral basis of the profession and professionals. However, this also requires fluent interaction within health care organisations (Waterfield & Barnason, 2022), but also paying attention to working conditions, care, and working environments (Aronsson et al., 2017), with careful prevention of stress, including moral distress. Teamwork and sharing responsibilities have previously been seen as supporting factors for moral competence in nurses (Wiisak et al., 2024), which can be developed with the support of nurse managers who have also been educated on ethics and ethical leadership.

At the societal level, support for nurses’ moral competence is important. It can be supported through national or international health policy documents and ethical guidelines promoted not only by nursing boards but mainly by other decision‐makers—at the health policy level. In addition, social recognition was seen as a fundamental part of support for nurses’ moral competence. Having a sense of societal appreciation and personal calling have been identified as key factors that influence nurses to remain in the profession, but they are also motivational factors for joining nursing (McKenna et al., 2023).

A wide range of procedures, structures and interventions, in addition to basic university education, could be used in an organisation to promote the consideration of ethical issues. Ethical issues arise from conflicting values and are complex in nature (Haahr et al., 2020). Inevitably, there is no single right solution, and conflicts of values require a multifaceted approach, principles‐based decision‐making and debate (Kälvemark et al., 2004). Sensitivity to ethical issues and motivation to explore ethical issues in care arise from the competence of individuals and are diversified by the mutual desire of the group to learn and seek justification for different situations. Ethical questions are not positive or negative in nature, but situations in which one must reflect on what is good or bad, promote the good of the patient, and avoid harm are inevitable. Since ethical issues also occur at the level of professionals, at the organisational level and even at the level of society, various means, methods, and organisational structures are needed to support moral competence and the consideration of ethical issues. In addition, public awareness of the role of nurses and the ethical perspective of the profession has been raised, especially after the COVID‐19 pandemic.

## CONCLUSION AND RECOMMENDATIONS

Moral competence is part of professional competence. It forms a continuum, from its sensitisation to its advanced development, passing through different stages where an integrated multifaceted support strategy is required. Nurses’ moral competence could benefit from continuous support from colleagues, superiors, organisations, and society. Moreover, easy‐to‐use and practical tools and approaches, such as ethics protocols, toolkits, and education, could be profitable.

## IMPLICATIONS

The study provides knowledge on how nurses’ moral competence can be supported. This knowledge may inform nursing practice, management, policy‐making, research, and education. The establishment of both national‐ and international‐level ethics indicators for health policy is needed to ensure ethically sustainable care. In addition, there is a need for investment in and the establishment of strategies and regulations on ethics management in health systems to support nurses’ moral competence. This study demonstrates that several practices, interventions, and procedures exist that need to be implemented to support nurses’ moral competence. For research, the findings suggest that complex interventions are needed to support nurses’ moral competence as it is multilevel, cross‐cutting different individual, organisational, and societal levels. Multiple stakeholders could be involved in future research aiming at assessing the effectiveness of specific interventions in the capacity to support nurses and prevent, for example, moral distress. For education, designing and offering consistent educational strategies at the undergraduate and postgraduate university levels, as well as continuous education and professional development, is needed to support nurses’ moral competence.

## AUTHOR CONTRIBUTIONS

*Study design*: Riitta Suhonen, Chris Gastmans, Brian Keogh, Evridiki Papastavrou, and Alvisa Palese. *Data collection*: Johanna Wiisak, Riitta Suhonen, Alessandro Galazzi, Chris Gastmans, Brian Keogh, Evridiki Papastavrou, Alvisa Palese, and Minna Stolt. *Data analysis*: Alessandro Galazzi, Nikos Stefanopoulos, Alvisa Palese, Minna Stolt. *Study supervision*: Alvisa Palese, Minna Stolt. *Manuscript writing*: Johanna Wiisak, Riitta Suhonen, Alvisa Palese, and Minna Stolt. *Critical revisions for important intellectual content*: Chris Gastmans, Brian Keogh, Evridiki Papastavrou, Nikos Stefanopoulos, and Alessandro Galazzi.

## CONFLICT OF INTEREST STATEMENT

The author(s) declared no potential conflicts of interest with respect to the research, authorship, and/or publication of this article.

## ETHICS STATEMENT

The study was approved by the Ethical Committees of four countries (Cyprus, EC 2023.01.82; Greece, EC 29192/11‐4‐23; Ireland EC 230501; Italy, IRB 33/2023). According to legislation in two countries (Belgium and Finland), ethical approval in this kind of study was not required; however, in Finland, the study was approved by the Internal Review Board (Finland, IRB 18042023).

## Supporting information



Supporting Information

Supporting Information
