# Supplementary material for: How nurses’ moral competence can be supported: Findings from international focus groups with professionals
Source: Int Nurs Rev. 2024 Nov 27;72(3):e13080. doi: 10.1111/inr.13080 (PMC12206691; doi:10.1111/inr.13080)
Supplement: Supplementary file 1 — Supporting Information [file INR-72-0-s002.docx]

**Supplementary File 1.** COnsolidated criteria for REporting Qualitative research checklist (COREQ), Tong et al., 2007.

| **Topic, Item No.** | | **Guide Questions/Description** | | | **Brief description** |
| --- | --- | --- | --- | --- | --- |
| **Domain 1: Research team and reﬂexivity** | | | | | |
| *Personal characteristics* | | | | | |
| Interviewer/facilitator | 1 | | Which author/s conducted the interview or focus group? | Two researchers in each country as moderator and observer. Moderators lead the discussion according to the research protocol; the observer made field notes about discussion, persons involved and social interaction within the group | |
| Credentials | 2 | | What were the researcher’s credentials? e.g. PhD, MD | All were educated at the Master of Science or PhD level | |
| Occupation | 3 | | What was their occupation at the time of the study? | The researchers were appointed at the university level with different roles, as reported in the affiliations of the authors and members of the PROMOCON* consortium | |
| Gender | 4 | | Was the researcher male or female? | There were involved both males and females | |
| Experience and training | 5 | | What experience or training did the researcher have? | All researchers had previous experience in designing and conducting focus groups | |
| *Relationship with participants* | | | | | |
| Relationship established | 6 | | Was a relationship established prior to study commencement? | No personal relationship between researchers and participants were established prior to the study commencement | |
| Participant knowledge of  the interviewer | 7 | | What did the participants know about the researcher? e.g. personal goals, reasons for doing the research | Participants were informed regarding their background and the research interests of researchers; there were no friendships established before the study commencement | |
| Interviewer characteristics | 8 | | What characteristics were reported about the interviewer/facilitator?  e.g. Bias, assumptions, reasons and interests in the research topic | Interviewers presented themselves as members of the project and as researchers of the university involved in the project | |
| **Domain 2: Study design** | | | | | |
| *Theoretical framework* | | | | | |
| Methodological orientation and Theory | 9 | | What methodological orientation was stated to underpin the study? | A qualitative descriptive study design was used | |
| *Participant selection* | | | | | |
| Sampling | 10 | | How were participants selected? e.g. purposive, convenience,  consecutive, snowball | A stratified purposive sampling method was adopted | |
| Method of approach | 11 | | How were participants approached? e.g. face-to-face, telephone, mail,  email | Participants were contacted by telephone and email. An information brochure was also prepared with a detailed explanation of the (a) purposes of the PROMOCON* project; (b) the aims of the study; (c) the description of the role and rights of the participants, and (d) contact details of the research team | |
| Sample size | 12 | | How many participants were in the study? | From 5 to 8 in each focus group per country (namely Belgium, Cyprus, Finland, Greece, Ireland and Italy) | |
| Non-participation | 13 | | How many people refused to participate or dropped out? Reasons? | Participation was voluntary therefore no data about refusal is available. | |
| Setting | | | | | |
| Setting of data collection | 14 | | Where was the data collected? e.g. home, clinic, workplace | Focus groups took place in a sufficiently large, private room in the university departments where the researchers work, and where there was no disturbance from ambient noise | |
| Presence of non-  participants | 15 | | Was anyone else present besides the participants and researchers? | None was present | |
| Description of sample | 16 | | What are the important characteristics of the sample? e.g. demographic  data, date | A socio-demographic form was filled in by participants. Their main characteristics are reported in the findings section | |
| *Data collection* | | | | | |
| Interview guide | 17 | | Were questions, prompts, guides provided by the authors? Was it pilot tested? | An interview guide was developed by one country of the PROMOCON* project members and pilot tested by the one country. All partners followed this guide. The focus groups were conducted in the native language of participants. | |
| Repeat interviews | 18 | | Were repeat inter views carried out? If yes, how many? | No repeated interviews were performed | |
| Audio/visual recording | 19 | | Did the research use audio or visual recording to collect the data? | All focus group were audio recorded after having obtained the written informed consent from each participant, and the recordings of the interviews were transcribed verbatim in the local language by one researcher | |
| Field notes | 20 | | Were field notes made during and/or after the interview or focus group? | Field notes were made by the observers during focus groups however, these were not considered in this data analysis | |
| Duration | 21 | | What was the duration of the interviews or focus group? | All focus group discussions lasted approximately more than two hours; there was no break during the meeting, as the participants indicated that it was not necessary | |
| Data saturation | 22 | | Was data saturation discussed? | Data saturation was discussed during the translational data analysis process | |
| Transcripts returned | 23 | | Were transcripts returned to participants for comment and/or correction? | Transcripts were not returned to the participants to comments and/or correction | |
| **Domain 3: analysis and ﬁndings** | | | | | |
| *Data analysis* | | | | | |
| Number of data coders | 24 | | How many data coders coded the data? | From two to three researchers coded the data at the national level, while at the international level, five researchers were involved, at least one for each country in other to ensure representativeness | |
| Description of the coding  tree | 25 | | Did authors provide a description of the coding tree? | At the national level, the coding three was created in the national language that is available from authors upon request | |
| Derivation of themes | 26 | | Were themes identified in advance or derived from the data? | The main research questions guided the data analysis: there were used both a deductive and an inductive approach as summarized in detail in the data analysis section of the manuscript | |
| Software | 27 | | What software, if applicable, was used to manage the data? | The data analysis at the country and at the translational levels was performed manually and no software was used | |
| Participant checking | 28 | | Did participants provide feedback on the findings? | Participants did not provide feedback on the findings | |
| *Reporting* |  | |  |  | |
| Quotations presented | 29 | | Were participant quotations presented to illustrate the themes/findings?  Was each quotation identified? e.g. participant number | The anonymized quotes taken from the transcripts were included in the results to illustrate the identified themes and sub-themes. The quotes were left at the country level, without indicating the participant number identification which is available from authors | |
| Data and findings consistent | 30 | | Was there consistency between the data presented and the findings? | The consistency of the data and the findings has been ensured as reported in the data analysis and in the findings | |
| Clarity of major themes | 31 | | Were major themes clearly presented in the findings? | Major themes were presented in the findings in a narrative manner and summarized in tables with quotes | |
| Clarity of minor themes | 32 | | Is there a description of diverse cases or discussion of minor themes? | Given the complexity of the analysis, as well as the process used, only those themes saturating the data across countries were reported | |

*PROmoting a MOrally COmpetent Nurse (PROMOCON)

Developed from: Tong A, Sainsbury P, Craig J. Consolidated criteria for reporting qualitative research (COREQ): a 32-item checklist for interviews and focus groups. International Journal for Quality in Health Care. 2007. Volume 19, Number 6: pp. 349 – 357.
