# Supplementary material for: How nurses’ moral competence can be supported: Findings from international focus groups with professionals
Source: Int Nurs Rev. 2024 Nov 27;72(3):e13080. doi: 10.1111/inr.13080 (PMC12206691; doi:10.1111/inr.13080)
Supplement: Supplementary file 2 — Supporting Information [file INR-72-0-s001.docx]

**Supplementary File 2. Focus group protocol and interview guide**

**Methods**

**Design:**

Qualitative research approach using focus group interviews will be employed. A focus group is, according to Lederman (see Thomas et al. 1995), ‘a technique involving the use of in-depth group interviews in which participants are selected because they are a purposive, although not necessarily representative, sampling of a specific population, this group being ‘focused’ on a given topic’. Focus group is unique having an ability to generate data based on the synergy of the group interaction (Green & Thorogood 2004).

**Sample and access to the field:**

Inclusion criteria for the interview participants:

1. undergraduate nursing students
2. post-graduate nursing students (e.g. masters and doctors)
3. professional nurses who have a nursing qualification (registered nurse)
4. potential nursing educators as master’s and doctoral students (whose intent is to undertake the career as educators)

Exclusion criteria:

1. professionals not willing to participate
2. not active in their professional or student role

One focus group with four to nine (max.) participants from each of the six participating countries will be recruited.

Field access:

- Purposeful sampling will be used, recruiting through the responsible regional coordinators (= team representative) in the partner countries

**Data collection:**

Data will be collected using focus group interviews (voluntariness/data protection).

- Two interviewers (interview transcript), moderator and two observer
- Interview language: preferably participants’ native language (multinational focus groups in English)
- Digital communication programme (Zoom or Teams or similar) / if onsite: Seated in a circle (create a drawing with seat numbers)
- Audio recording of the interview and filed notes of the main replies incl. seat number (intensity of participation in group discussion)
- Digital surroundings (note informed consent) / Quiet room, no visual or acoustic distractions
- Finally: the various competencies are written down on small cards and then rated by consensus method (the participants make order for identified competencies referring to ethics) and the result will be documented by a photo.

Interviewer qualifications:

- Experience in conducting qualitative interviews
- No current or previous interdependency to the interview participants

**Data transfer and data analysis:**

- Documented summary of the audio recordings in English (protocol focusing on the questions in the guideline)
- Photographic documentation of the prioritization of the needs and contents for developing ethical competencies of nurses and translation into English
- Summarizing qualitative content analysis of the audio protocols; structuring according to main and side issues (deductive) taking any new impulses into consideration (deductive) (Krueger 1994)
- Consensus within the focus groups – written main themes

**Basic data to be collected:**

- In each country **nurse professionals** working in macrolevel (hospital, long-term care, home care) and micro level (ward or unit)
  - Age (in years):
  - Gender:
  - Highest school qualification:
  - Highest nursing qualification:
  - Additional professional nursing qualification:
  - Years of nursing experience:
  - Main role responsibilities

Table 1. Interview guide

| **Interview phase** | **Target/Function** | **Theme** | ***Question to the focus group*** | ***Follow-up question(s)*** | **Notes** |
| --- | --- | --- | --- | --- | --- |
| *The arrangements should ensure a comfortable conversation atmosphere. Comfortable chairs and the offer of coffee and light pastries increase the comfort feeling.* | | | | | |
| Opening  (2 minutes) | To guarantee the comprehensibility and to ensure the course of the interview | Welcome and instructions | *Thank you very much for agreeing to take part in the interview. I shall be recording the conversation so that I can concentrate better on your replies.*  *In order to be able to take all your remarks into consideration, it is important that you speak* ***one after the other****. If necessary, I will remind you about this.* | | Ensure a relaxed atmosphere |
| Introduction to the subject  (5 minutes) | Clarify the preconceptions of the interview partners. | Definitions of ethical (Moral) competence | *Describe how you think a nurse professional (for example, a registered nurse – for future nurse), with excellent ethical competence should be!* | *What do you mean by a ethically competent nurse?*  *How would you define an “excellent ethically competent nurse”?* | If necessary, use the problem reversal method (What do you mean by a bad leader?) |
| Transition  (5-15 minutes) | Present your own experiences. | Exchange of experiences. | *What experiences have you had regarding ethical competence of nurses?* | *What example can you give?*  *Which situation would you like to describe?*  *Which key figures do you have in your mind’s/ eye?* | Prevent verbosity  If necessary, move to the next question; protect the anonymity of the key figures. |
|  | Show the contrasts between the experiences. |  | *That all sounded very positive/negative.*  *What other, contrary experiences have you had?* | |  |
| Key questions  (25-50 minutes) | **Map out ethically demanding situations that require high ethical competence of nurses / future nurses.** | Ethically difficult situations in clinical nursing practice | *Which ethically demanding situations can you describe that you have experienced or witnessed in clinical nursing practice?* | *Which ethical competencies would help meet the increasing demands in clinical nursing practice?* |  |
|  | **Map out ethical/ moral competencies nurses / future nurses need in providing ethically high-level clinical nursing care.** (profiles will show the levels) | Ethical competencies as knowledge | *In your opinion, what kind of knowledge about ethical issues nurses need in clinical nursing practice?* | *What nurses’ knowledge areas related to ethics need to be developed?* |  |
| **Interview phase** | **Target/Function** | **Theme** | ***Question to the focus group*** | ***Follow-up question(s)*** | **Notes** |
|  |  | Ethical competencies as skills and behaviour | *In your opinion, what kind of skills / behaviors nurses need in clinical nursing practice to act in ethically high level?* | *What nurses’ skills/ behaviors areas need to be developed?* |  |
|  |  | Ethical competencies as attitudes | *In your opinion, what kind of attitudes nurses need to have in clinical nursing practice to act in ethically high level?* | *What nurses’ attitudes need to be developed?* |  |
|  |  | Ethical competencies as performance | *In your opinion, what kind of nurses’ performance is needed in providing ethically high-level nursing care?* | *What nurses’ performance areas need to be developed to provide ethically/ morally high-level care?* |  |
| Key questions (continued) | **Map out what methods may be used to develop ethical competence** | Role models, experiences, good examples, interventions | *In your opinion, which competencies of role models/supervisors/teachers have a positive effect on the attitudes of nursing students to act in ethically appropriate way (provide high-level nursing care)?* | *Which characteristics and behavior patterns of role models have an almost negative effect on the attitudes of nurses to act ethically appropriate way?* |  |
| **Interview phase** | **Target/Function** | **Theme** | ***Question to the focus group*** | ***Follow-up question(s)*** | **Notes** |
| Key questions (continued) | Specify competencies concerning identified fields of action. | Support for ethically competent nursing care | *In your opinion, how nurses can be supported to act in ethically high-level (provide ethically high-level nursing care)?* | *What is required for the*   - *colleagues?* - *superiors?* - *healthcare organisation?* - *society?* - *other stakeholders?* | Call for brainstorming |
| Summing up/ conclusion  (5-15 minutes) | Ranking of competencies with regard to nurses’ ethical competence | Forming a consensus Competence profile | *We have prepared some little cards with skills and competencies from the discussion. Would you please now in your group bring these into a rating list:*   1. *What are very important and extremely relevant skills/competencies for an educational program focusing on developing ethically competent nurses? (Stack A)* 2. *What are very important skills/competencies that, in your opinion, could not be developed well through education programs? (Stack B)* 3. *What skills/competencies are only of secondary importance for ethical competence development in clinical nursing practice? (Stack C)* 4. *In your opinion, what skills/competencies are unimportant for ethical competence development in clinical nursing practice? (Stack D)*   *You are welcome to add your own skills/competencies by using the blank cards. Please lay the cards next to each other so that we can photograph your rating lists properly.*  *You have 5 minutes time to form a consensus.*  *Thanks very much for creating these inspiring rating lists!* | | Make sure there is sufficient space on the table – platform.  Leave some cards blank for supplements and additions, and distribute if required.  Extend by 10 minutes, if required. |
| **Interview phase** | **Target/Function** | **Theme** | ***Question to the focus group*** | ***Follow-up question(s)*** | **Notes** |
| Wrapping up  (2-5 minutes) | Show appreciation and esteem, clarify outstanding questions. | Conclusion | *Before we now come to the end, I would like to know what else you find important about nurses’ ethical competence that was perhaps not (sufficiently) discussed during our talk?* | *What questions have still to be clarified?*  *Would you like to ask me anything else?* |  |
| End  (<1 minute) | Goodbyes | Thanks | *Then thank you very much, everyone, for the conversation and your frankness as well as the valuable impulses given. I wish you all the best! I’m now switching off the tape.* | |  |
